# Evaluation the static precise point positioning and fixing rate with phase ambiguity resolution using different analysis centers products

**DOI:** 10.1371/journal.pone.0322622

**Published:** 2025-05-19

**Authors:** Xingli Sun, Jinjie Yao, Zhiliang Yang

**Affiliations:** 1 School of Information and Communication Engineering, North University of China, Taiyuan, Shanxi, China; 2 Shanxi Province Key Laboratory of Intelligent Detection Technology & Equipment, North University of China, Taiyuan, Shanxi, China; University of Shanghai for Science and Technology, CHINA

## Abstract

The performance of precise point positioning with phase ambiguity resolution (PPP-AR) strongly depends on the quality of analysis centers (ACs) products. Nowadays, many IGS/MGEX ACs provide PPP-AR products. To give a full evaluation of PPP-AR performance with various products, this work comprehensively investigates the positioning performance and AR fixing rate of GPS-only and Galileo-only with a complete set of products using open-source software PRIDE PPP-AR. We analyze six products, including COD0_MGX_FIN, COD0_OPS_FIN, GBM0_MGX_RAP, GRG0_OPS_FIN, WUM0_MGX_RAP, and WUM0_MGX_FIN, from four ACs: CODE, GFZ, CNES, and WHU. Then, 7 days of GPS and Galileo data were collected from 42 globally distributed MGEX stations. The root mean square (RMS) value of GBM0_MGX_RAP product for GPS-only reached about 3 mm, 2 mm, 4 mm, and 6 mm in east, north, up, and 3D components, respectively. The other five products have reached about 2 mm, 2 mm, 4 mm, and 5 mm, respectively. Compared to other products, the narrow-lane (NL) fixing rate of the GBM0_MGX_RAP product is about 20% lower for GPS-only. The Galileo-only positioning using four products (except COD0_MGX_FIN and GBM0_MGX_RAP) reached about 2 mm, 1.9 mm, 5.8 mm, and 6.4 mm in east, north, up, and 3D components, respectively, COD0_MGX_FIN reached 2.4 mm, 2.2 mm, 5.9 mm, and 6.7 mm, respectively. GBM0_MGX_RAP reached about 3.5 mm, 3.0 mm, 8.5 mm, and 9.7 mm, respectively. The wide-lane (WL) and NL fixing rate of Galileo-only showed five products have all exceeded 99%, the GBM0_MGX_RAP product is 97.24% and 92.56%, respectively. Overall, COD0_OPS_FIN, WUM0_MGX_RAP, and WUM0_MGX_FIN products rank first, COD0_MGX_FIN and GRG0_OPS_FIN take the second place, while those with the GBM0_MGX_RAP shows the worse performance.

## 1. Introduction

Precise point positioning (PPP) is a method of achieving high-precision positioning by using a single Global Navigation Satellite System (GNSS) receiver [1]. It integrates the technical advantages of standard single-point positioning and relative positioning, and is another technological revolution after the GNSS positioning technology relays the real-time kinematic (RTK) positioning technology [2]. The PPP is used in many engineering applications and scientific research fields, such as high-precision static and kinematic positioning, precise timing, precise orbit determination of low-orbit satellites, tidal and sea level monitoring, water vapor remote sensing, water vapor inversion and ionospheric monitoring, seismic monitoring, and earth plate movement monitoring, because of its centimeter or millimeter level positioning, no limits on operating distance, flexible operation, and many other significant advantages [3–7]. However, due to the existence of phase biases (or uncalibrated hardware delays, or uncalibrated phase delay (UPD)), it’s difficult for traditional float PPP to reach its highest precision through ambiguity resolution (AR). Phase biases are originated from both receiver and satellite hardware and they’re coupled with undifferenced ambiguities in PPP [2,8]. Thus, unlike double-differenced RTK relative positioning ambiguities, PPP ambiguities lose their integer nature. As a result, PPP generally exhibits lower precision, especially in the east component, and a much longer convergence time than RTK relative positioning [2,9].

Regarding accuracy and convergence time, PPP with ambiguity resolution (PPP-AR) shows significantly better results than the ambiguity-float PPP [10–13]. Many scholars have conducted research on improving the positioning performance of PPP, such as the AR subset selection method [14], PPP integrated with inertial navigation system (INS) [15], PPP stochastic model estimation [16], and multi-frequency and multi-constellation PPP [17,18]. With the increasing attention of many scholars to PPP/PPP-AR, many research institutions have developed software for computing PPP products [19–24]. Fortunately, the satellite phase biases are found to be spatially and temporally stable, which means observations collected from different receivers at the same time contain the same phase biases for a satellite. The satellite-related part of phase biases must be computed from a global network to recover the integer nature of PPP ambiguities [8,25,26]. The receiver phase biases at a single station can be eliminated through between-satellite differences. Thus, PPP-AR can be fulfilled. There are several methods to fix the undifferenced ambiguity: integer clock model (IRC), decoupled clock model, UPD model, and phase clock/bias model [26–29]. The theoretical equivalence of the different PPP-AR methods has been proved [30–32], despite using various strategies in different products.

Moreover, the international GNSS service (IGS) and multi-GNSS experiment (MGEX) analysis centers(ACs) have started to release GPS, Galileo, BDS-2, and BDS-3 phase biases [31,33,34]. To ensure the interoperability of bias products from ACs, the observable-specific signal biases (OSBs) framework, instead of differential code bias (DCB) or UPDs or wide-lane (WL) and narrow-lane (NL) conventions, is preferred by the IGS [35]. OSBs mean that each specific code and carrier phase has a bias correction about each tracking channel of each satellite, and users simply need to subtract these biases from the raw measurements to carry out undifferenced GNSS PPP-AR processing [36]. Schaer and several other scholars released standard code OSB format bias-solution independent exchange format (SINEX) 1.00, and many ACs have provided code bias products in the OSB framework; next, they further proposed that phase bias can also be unified in Bias-SINEX 1.00 format [37–39]. The OSB framework has good interoperability such that the phase biases from different AR methods can be accommodated. The ACs of Center for Orbit Determination in Europe (CODE), Deutsches GeoForschungs Zentrum (GFZ), Centre National d’Etudes Spatiales and Collecte Localisation Satellites (CNES/CLS), and Wuhan University (WHU) have provided PPP-AR services for phase biases daily products in Bias-SINEX 1.00 format.

The ACs of CODE, GFZ, CNES/CLS, and WHU all provide a complete set of PPP-AR products for GPS and Galileo. For users who are unsure which IGS ACs’ PPP-AR product to choose when achieving high-precision positioning. Therefore, the purpose of this study is to assess the static positioning and fixing rate performance in 24-hour observing sessions, as derived from single-system PPP-AR solutions (i.e., GPS-only and Galileo-only)with six precise products from four ACs. There are different open-source software and online free PPP services for GNSS data processing with the PPP technique with/without ambiguity resolution in static and kinematic modes [40,41]. The research results of the literature indicate that PRIDE PPP-AR software performs the most precise option for positioning via static PPP of all open-source software and online PPP services [41].

This article selects open-source software PRIDE PPP-AR to evaluate the positioning and AR fixing rate performance of different IGS/MGEX products in static modes. We start with a brief presentation of the IGS/MGEX PPP-AR products and the functional model of GNSS users PPP-AR adopted in this work. Following that, the experimental dataset and data processing strategy are described. Afterward, the results of PPP-AR with various products are compared. Next, the network solution model was discussed. Finally, our study ends with conclusions.

## 2. Materials and methods

### 2.1. Overview of IGS/MGEX PPP-AR products

The precise products are indispensable for PPP-AR solutions. A few ACs of IGS/MGEX have generated ambiguity resolution daily products for users using different approaches to realize PPP-AR. The key difference of the product generation approaches is how the satellite phase biases are separated from the integer ambiguities. For more detailed information about the network solution methods, the reader is referred to [42,43]. Since the network solution is not within the scope of this study, the author will not elaborate further here.

Since the IGS/MGEX have begun to release GNSS phase bias products in recent years, users can introduce them along with the legacy satellite clock products into PPP to enable PPP-AR [38,43]. GNSS orbits, clocks, code, phase biases, and other products are generally computed based on observations of the IGS/MGEX tracking network and, optionally, other proprietary stations. To implement PPP-AR using these products, the corresponding precise GNSS orbit, clock, and bias products should be mix-wise used which ensures to be consistency with the server. Different products from different ACs cannot be mixed, even if they are products of different types from the same AC. Table 1 shows the IGS/MGEX ACs’ PPP-AR products provided daily products as of now, and the fields in Table 1 use the format used in long filenames. The sampling rate of orbit, clock, code, phase biases, earth orientation parameter (ERP), and attitude product files are different.

**Table 1:** Overview of the IGS/MGEX ACs’ PPP-AR products.

Institution	Product number	System	Sampling rate of orbit/clock/ code and phase biases/ERP/ attitude
CODE	COD0_MGX_FIN	G/R/E/C/J	5min/30s/1D_OSB/1D_12H/30s
	COD0_OPS_FIN	G/R/E	5min/30 s or 5s/1D_OSB/7D_1D/30s
GFZ	GBM0_MGX_RAP	G/E/C	5min/30s/1D_OSB/1D_1D/30s
CNES/CLS	GRG0_OPS_FIN	G/E	5min/30s/1D_OSB/7D_1D/30s
WHU	WUM_MGX_RAP	G/R/E/C	1min/30s/1D_ABS/1D_1D/30s
	WUM_MGX_FIN	G/R/E/C/J	1min/30s/1D_ABS/1D_1D/30s

The letters G, R, E, C, and J, denote, respectively, GPS, GLONASS, Galileo, BDS, and QZSS. The same letters will be used below. In the interest of brevity, COD0_MGX_FIN, COD0_OPS_FIN, GBM0_MGX_RAP, GRG0_OPS_FIN, WUM0_MGX_RAP, and WUM0_MGX_FIN denote PPP-AR products from CODE, GFZ, CNES/CLS, and WHU ACs, respectively, throughout the rest of this article, if there is no additional explanation given. All four ACs shown in Table 1 provide GPS and Galileo PPP-AR products. Starting from November 30, 2022 (i.e., day of the Year (DOY) 331, GPS week 2238) and later, the long product filenames apply to all IGS products [44]. For the fields and abbreviations in Table 1, please refer to the guidelines for long product filenames in the IGS [45,46].

The orbit exchange format (ORBEX) proposed at the 2017 IGS AC workshop is described [47,48]. Its main purpose is to provide flexibility in the exchange of satellite-related information considering the emergence of new constellations and additional types of satellite metadata [43]. Then IGS proposed that each AC should release attitude products that are compatible with clock deviation products, so that the satellite attitude models on both the user and server sides are consistent, thereby further improving the positioning accuracy of satellites during the shadow period [47,48]. Six different products, namely COD0_MGX_FIN, COD0_OPS_FIN, GBM0_MGX_RAP, GRG0_OPS_FIN, WUM0_MGX_RAP, and WUM0_MGX_FIN, from four ACs, CODE, GFZ, CNES/CLS, and WHU, were selected for comparative analysis.

This work comprehensively investigates the positioning performance of GPS-only and Galileo-only PPP-AR with the six precise products. COD0_OPS_FIN clock product files with the 30s or 5s sampling. Researchers have shown that, in static mode observations at rates higher than 30s do not improve the precision of the solution significantly [41]. To maintain comparability between different products, this article only analyzes clock products with a sampling rate of 30s.

All ACs products can be downloaded on their official website and the products are stored in weekly directories. After you successfully register, other products can also be downloaded on CDDIS’s official website (https://cddis.nasa.gov/archive/gnss/products/) [49], except for WUM0_MGX_RAP products. WUM0_MGX_RAP products at Wuhan University can be freely downloaded from (http://www.igs.gnsswhu.cn/) [50].

### 2.2. PPP model

In this section, we briefly introduce the PPP model. We also provide details on the processing strategy and the accuracy evaluation with emphasis on the single constellation approach. The undifferenced and uncombined functional model for pseudorange and carrier phase observations between the receiver and the satellite can be written as follows [33,51–53]:


$$\begin{array}{cc} & P_{\text{r},j}^{\left(s\right)}(t)=\rho_{\text{r}}^{\left(s\right)}(t-\tau ,t)+c(\delta t_{r}(t)-\delta t^{\left(s\right)}(t-\tau ))+I_{r,j}^{\left(s\right)}(t)+T_{r}^{\left(s\right)}(t)+d_{r,j}-d_{j}^{\left(s\right)}+\varepsilon_{P,j}^{\left(s\right)}(t)\\ & L_{\text{r},j}^{\left(s\right)}(t)=\rho_{\text{r}}^{\left(s\right)}(t-\tau ,t)+c(\delta t_{r}(t)-\delta t_{(}^{s)}(t-\tau ))-I_{r,j}^{\left(s\right)}(t)+T_{r}^{\left(s\right)}(t)+\lambda_{j}N_{r,j}^{\left(s\right)}+b_{r,j}-b_{j}^{\left(s\right)}+\varepsilon_{L,j}^{\left(s\right)}(t) \end{array}$$ (1)

where the subscripts r and j indicate the receiver and carrier frequency number, the superscript (s) identifies a GNSS satellite; $P_{r,j}^{\left(s\right)}(t)$ is observed pseudorange in j-th frequency at time t in units of meters; $L_{\text{r},j}^{\left(s\right)}(t)$ is observed carrier phase in j-th frequency at time t in units of meters;$\rho_{u}^{\left(s\right)}(t-\tau ,t)$ is the geometric range between position of satellite antenna phase center at time t−τ of signal transmission and position of receiver antenna phase center at time t of signal reception in units of meters; $\delta t_{r}(t)$ and $\delta t^{\left(s\right)}(t-\tau )$ are receiver clock biases at time t and satellite clock biases at time t−τ in units of seconds, respectively; c demotes the speed of light in vacuum in units of meters/second; $T^{\left(s\right)}(t)$ denotes the slant tropospheric delay in units of meters; $I_{r,j}^{\left(s\right)}(t)$ is the ionospheric delay on j-th frequency in units of meters; $\lambda_{j}$ denotes the carrier wavelength of j-th frequency in units of meter. $N_{\text{r},j}^{\left(s\right)}$ is the integer phase ambiguity in units of cycle $d_{r,j}\;and\;d_{j}^{\left(s\right)}$ denote the pseudorange bias of the j-th frequency,which is caused by the hardware delay of the receiver and the satellite pseudorange; $b_{r,j}$ and $b_{j}^{\left(s\right)}$ denote the carrier phase bias of the j-th frequency, which is caused by the hardware delay of the receiver and the satellite carrier;$\varepsilon_{P,j}^{\left(s\right)}(t)$ and$\varepsilon_{L,j}^{\left(s\right)}(t)$ are unmodelled error such as observation noise and multipath effect pseudorange and carrier phase respectively. It should be noted that equation 1 does not include satellite and receiver antenna phase center correction, relativistic effects, tidal load deformation (solid tide, polar tide, and sea tide), Sagnac effect, satellite antenna phase entanglement (only for carrier observation values), and other corrections, which have been corrected through the model in advance [54]. For simplicity, high-order ionospheric delay and multipath effect are omitted.

The ionosphere is one of the biggest error sources in GNSS PPP and navigation. For the first-order ionospheric delay in equation 1, can be eliminated by the difference between the product of the dual-frequency observations and their frequency squares. The GNSS i-th frequency and j-th frequency ionosphere-free (IF) observation equation is then formed [31,51,52]:


$$\begin{array}{cc} & P_{\text{r},IF}^{\left(s\right)}(t)=\alpha_{ij}P_{\text{r},i}^{\left(s\right)}(t)-\beta_{ij}P_{\text{r},j}^{\left(s\right)}(t)\\ & =\rho_{\text{r}}^{\left(s\right)}(t-\tau ,t)+c(\delta t_{r}(t)-\delta t_{(}^{s)}(t-\tau ))+T^{\left(s\right)}(t)+d_{r,IF}-d_{IF}^{\left(s\right)}+\varepsilon_{P,IF}^{\left(s\right)}(t)\\ & L_{\text{r},IF}^{\left(s\right)}(t)=\alpha_{ij}L_{\text{r},i}^{\left(s\right)}(t)-\beta_{ij}L_{\text{r},j}^{\left(s\right)}(t)\\ & =\rho_{\text{r}}^{\left(s\right)}(t-\tau ,t)+c(\delta t_{r}(t)-\delta t_{(}^{s)}(t-\tau ))+T^{\left(s\right)}(t)+\lambda_{IF}N_{\text{r},IF}^{\left(s\right)}+b_{r,IF}-b_{IF}^{\left(s\right)}+\varepsilon_{L,IF}^{\left(s\right)}(t) \end{array}$$ (2)

where$\begin{array}{cc} & \alpha_{ij}=\frac{f_{i}^{2}}{f_{i}^{2}-f_{j}^{2}}\\ & \beta_{ij}=\frac{f_{j}^{2}}{f_{i}^{2}-f_{j}^{2}} \end{array}$

and


$$\begin{array}{cc} & \lambda_{IF}N_{\text{r},IF}^{\left(s\right)}=\alpha_{ij}\lambda_{i}N_{\text{r},i}^{\left(s\right)}-\beta_{ij}\lambda_{j}N_{\text{r},j}^{\left(s\right)}\\ & d_{r,IF}=\alpha_{ij}d_{r,i}-\beta_{ij}d_{r,j}\\ & d_{IF}^{\left(s\right)}=\alpha_{ij}d_{i}^{\left(s\right)}-\beta_{ij}d_{j}^{\left(s\right)}\\ & b_{r,IF}=\alpha_{ij}b_{r,i}-\beta_{ij}b_{r,j}\\ & b_{IF}^{\left(s\right)}=\alpha_{ij}b_{i}^{\left(s\right)}-\beta_{ij}b_{j}^{\left(s\right)} \end{array}$$

where $P_{\text{r},IF}^{\left(s\right)}(t)$ and $L_{\text{r},IF}^{\left(s\right)}(t)$ are the IF GNSS pseudorange observation and carrier phase observation,respectively. Correspondingly, $d_{r,IF}$ and $d_{IF}^{\left(s\right)}$ are the ionosphere-free pseudorange bias at the receiver end and the satellite end, $b_{r,IF}$ and$b_{IF}^{\left(s\right)}$ are the ionosphere-free combination phase bias at the receiver end and the satellite end respectively. $\lambda_{IF}$ is the wavelength of GNSS IF combination, $N_{\text{r},IF}^{\left(s\right)}$ is IF ambiguity term. The IF combination eliminates the first-order term of ionospheric delay and is the most commonly used observation model in PPP. Please refer to references [31,51,52]. for the modeling method of PPP-AR for the tropospheric delay.

### 2.3. PPP-AR model

From (2), it can be seen that the GPS and Galileo IF observations contain the code and phase biases related to both the receiver and satellite. These biases will destroy the integer property of the carrier phase ambiguity if they are not handled properly. For the traditional satellite precise products that provided by the IGS, the satellite clock offsets assimilate the satellite code bias. And in the conventional GPS PPP user model, the receiver code bias is absorbed by receiver clock error. Thus, the ambiguity parameter, the receiver code bias, the receiver phase bias, the satellite code bias, and the satellite phase bias are lumped together and they cannot be separated from each other.

The traditional single difference (SD) ambiguity fixing method eliminates phase deviation at the receiver end by pairwise differentiation between different satellites and obtains single difference ambiguity with integer characteristics. Due to the difficulty in directly fixing the combination ambiguity without ionosphere, it is usually decomposed into WL and NL with sequentially fixed ambiguity. Among them, WL ambiguity can be obtained through the combination of IF and geometric distance free (HMW), while NL ambiguity can be calculated through integer WL ambiguity and IF combination ambiguity. After restoring the integer characteristics of single difference ambiguity, constraints are applied to the normal equation, ultimately achieving PPP-AR solution estimation.

The estimation parameters of GNSS PPP-AR contain the station coordinate, tropospheric delay, the ambiguities of satellites, and the receiver phase clock offset. The procedures are summarized as follows [31,33,55]:

(1) The GNSS PPP-AR solutions are estimated on the basis of PPP float estimation parameters. So, GNSS PPP float solutions are estimated by Kalman filtering or Least Squares Estimation (LSE) firstly. Then, the float ambiguity $\Delta \overset{\mathrm{\backslash lower}0.5em\text{\textbackslash{}smash\{\textbackslash{}scriptscriptstyle\textbackslash{}smile}}{\}}N_{\text{r},IF}^{m,n}$ can be obtained after SD.


$$\Delta \overset{\mathrm{\backslash lower}0.5em\text{\textbackslash{}smash\{\textbackslash{}scriptscriptstyle\textbackslash{}smile}}{\}}N_{\text{r},IF}^{m,n}=N_{\text{r},IF}^{m}-N_{\text{r},IF}^{n}$$ (3)

where the subscripts m and n represent different satellites, Δ represents the SD results between-satellites.

(2) WL integer ambiguity resolution.

The SD between-satellites method is applied in Melbourne-Wübbena(MW) observation to eliminate the influence of receiver-related hardware delays. Then, the MW observation equation is obtained [31]:


$$\begin{array}{c} \begin{array}{cc} & \Delta L_{r,MW}^{m,n}=\frac{1}{\gamma^{2}-1}(\gamma^{2}\Delta L_{r,1}^{m,n}-\Delta L_{r,2}^{m,n})-\frac{1}{\gamma^{2}+1}(\gamma^{2}\Delta P_{r,1}^{m,n}-\Delta P_{r,2}^{m,n})\\ & \;\;=\Delta \rho_{M}^{W}+\lambda_{WL}(\Delta N_{r,WL}^{m,n}+\Delta b_{r,MW}^{m,n})+\zeta_{r,MW}^{m,n} \end{array} \end{array}$$ (4)

where $\gamma^{2}=\frac{f_{1}^{2}}{f_{2}^{2}}$

The WL ambiguity $\Delta N_{\text{r},IF}^{m,n}$ can be computed by a simple rounding method due to its relatively long wavelength.


$$\Delta N_{\text{r},WL}^{m,n}=round(\frac{\Delta L_{r,MW}^{m,n}-(\Delta \rho_{r,MW}^{m,n}+\lambda_{WL}\Delta b_{r,MW}^{m,n})}{\lambda_{WL}}+\frac{\zeta_{r,MW}^{m,n}}{\lambda_{WL}})$$ (5)

Factors affecting the fixed ambiguity of WL are correction of antenna phase center, WL phase deviation, and errors that have not been fully corrected.

(3) NL integer ambiguity resolution.

The SD NL float ambiguity $\Delta \overset{\mathrm{\backslash lower}0.5em\text{\textbackslash{}smash\{\textbackslash{}scriptscriptstyle\textbackslash{}smile}}{\}}N_{\text{r},NL}^{m,n}$ can be calculated by (6). Then, the NL ambiguity$\Delta N_{\text{r},NL}^{m,n}$ can be fixed as integer by means of the LAMBDA or LS method.


$$\lambda_{IF}\Delta N_{I}^{F}=\frac{1}{\gamma^{2}-1}(\gamma^{2}\lambda_{1}\Delta N_{r,1}^{m,n}-\lambda_{2}\Delta N_{r,2}^{m,n})=\lambda_{NL}\Delta N_{r,NL}^{m,n}+\frac{1}{\gamma^{2}-1}\lambda_{2}\Delta N_{r,WL}^{m,n}$$ (6)

$$\Delta N_{\text{r},NL}^{m,n}=round(\frac{\lambda_{IF}\Delta N_{r,IF}^{m,n}-\lambda_{2}\Delta N_{r,WL}^{m,n}/(\gamma^{2}-1)}{\lambda_{NL}})$$ (7)

_or_


$$\Delta N_{\text{r},NL}^{m,n}=LAMBDA(\Delta \overset{\mathrm{\backslash lower}0.5em\text{\textbackslash{}smash\{\textbackslash{}scriptscriptstyle\textbackslash{}smile}}{\}}N_{\text{r},NL}^{m,n})$$ (8)

(4) The integer ambiguities $\Delta N_{\text{r},WL}^{m,n}$ and $\Delta N_{\text{r},NL}^{m,n}$ will be introduced as constraints for the observation equations, and then, the PPP-AR solutions will be achieved by estimation.

The following Table 2 gives the L1, L2, WL and the NL wavelengths for GPS and Galileo frequencies used in this article.

**Table 2:** Different types of GPS and Galileo wavelengths (unit: m).

	$\lambda_{1}$	$\lambda_{2}$	$\lambda_{WL}$	$\lambda_{NL}$
**GPS**	0.190	0.244	0.862	0.107
**Galileo**	0.190	0.255	0.751	0.109

## 3. Data and processing strategies

This section describes the experimental setup based on observation data from the globally distributed MGEX tracking stations. The distribution of stations involved is depicted, and the data processing strategy is described.

### 3.1. Observation dataset

**Fig 1:**
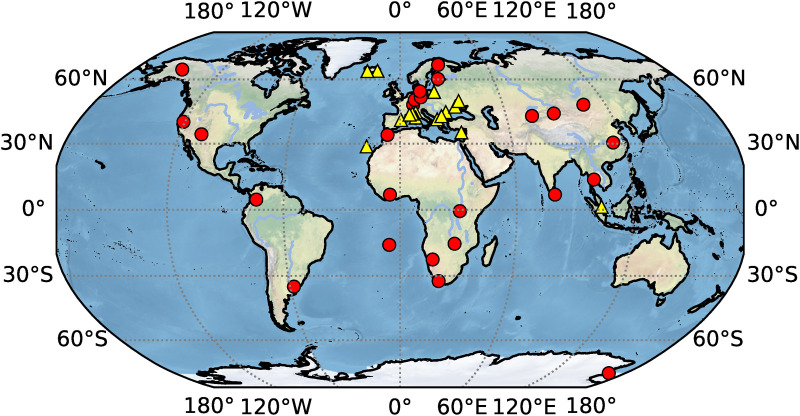
Geographic distribution of the 42 MGEX stations used in this study.

PPP-AR processes were performed for one week (DOY:155–161) in 2023 using 42 MGEX stations. The sampling interval of observations at these stations is 30s. All these MGEX stations can observe the observations of GPS and Galileo. GPS observations on L1 and L2 and Galileo observations on E1 and E5a are used to build the IF LC, and their geographical distribution is exhibited in Fig 1. The visualized global map is based on a Python program using the Matplotlib [56] and Cartopy [57] modules.

These stations are equipped with JAVAD or LEICA receivers. Among JAVAD receivers, some firmware versions of JAVAD TRE_3, JAVAD TRE_3 DELTA, and JAVAD TRE_3L DELTA. Among LEICA receivers, some firmware versions of LEICA GR30 and LEICA GR50. The red circle represents the JAVAD series receiver with a total of 26 stations, while the yellow triangle represents the LEICA series receiver with a total of 16 stations in Fig 1. The observation channels or codes of LEICA and JAVAD receivers are different. The specific L1 and L2 IF combinations of different receiver station observations are shown in Table 3.

**Table 3:** Different receiver IF channel or code combinations.

IF combinations	JAVAD	LEICA
**GPS**	1W/2W or1C/2X*	1C/2W
**Galileo**	1X/5X	1C/5Q

The firmware of the JAVAD receiver at HUEG station is JAVAD TRE_ 3L DELTA. Only the HUEG station is an L1W/L2W and L1C/L2X hybrid observation. The JAVAD receivers of the other 25 stations only observe the L1W/L2W IF combination observation. The GBM0_MGX_RAP products about GPS only have 1W/2W code and carrier phase bias products, the results of the 26 JAVAD series receivers stations will be analyzed. The GBM0_MGX_RAP products about Galileo only have 1C/5Q code and carrier phase bias products, the results of the 16 LEICA series receivers stations will be analyzed. COD0_MGX_FIN, COD0_OPS_FIN, GRG0_OPS_FIN, WUM0_MGX_RAP, and WUM0_MGX_FIN, these five PPP-AR products contain all the IF combinations of GPS and Galileo in Table 3, so the results of all 42 stations are analyzed.

### 3.2. Processing strategies

The PPP-AR calculations in this article were all completed on the open-source PRIDE PPP-AR software. The update time and version number of this software are 2023-08-07 and v2.2.6, respectively. The PRIDE PPP-AR software is an open-source software package based on many GNSS professionals’ collective work in the GNSS Research Center, Wuhan University [41,42,58,59]. The software package, user manuals, and more detailed information such as compatibility of GNSS constellations and signals can be downloaded from the PRIDE official website [60]. The PPP-AR processing strategy based on the default configuration and the PRIDE PPP-AR software is summarized in Table 4.

**Table 4:** PPP-AR processing parameters.

Item	Strategies
PPP modes	GPS Standalone, Galileo Standalone
Frequencies	G: L1/L2; E: E1/E5a
Estimator	Least Squares estimator
Data pattern	strict editing mode
Elevation mask	7°
ZTD model	piece-wise constant(PWC) is estimated in 60 min
HTG model	PWC is 720 min
PCOs and PCVs	Corrected with IGS20 atx file
Tides	Corrected according to IERS Conventions
Phase wind-up	Corrected
Ionospheric effect	Removed by IF linear combination
Tropospheric delay	Corrected with Saastamoinen model and global mapping function, and estimate residual zenith wet delays
Phase Ambiguities	Fix for G, E

## 4. Results

PPP-AR will output ITRF solutions in the IGS20 reference frame. This new reference frame is the IGS realization of ITRF2020. For more information on the IGS20 reference frame, please see the IGS20 release announcements [44]. The IGS daily solutions in solution independent exchange (SINEX) format were adopted as the external reference coordinates. The coordinates resulting from the processing with the six PPP-AR products referenced in ITRF20 for the 42 MAGX stations are transformed into ENU absolute coordinates for each day.

### 4.1. PPP-AR performance of GPS-only

**Fig 2:**
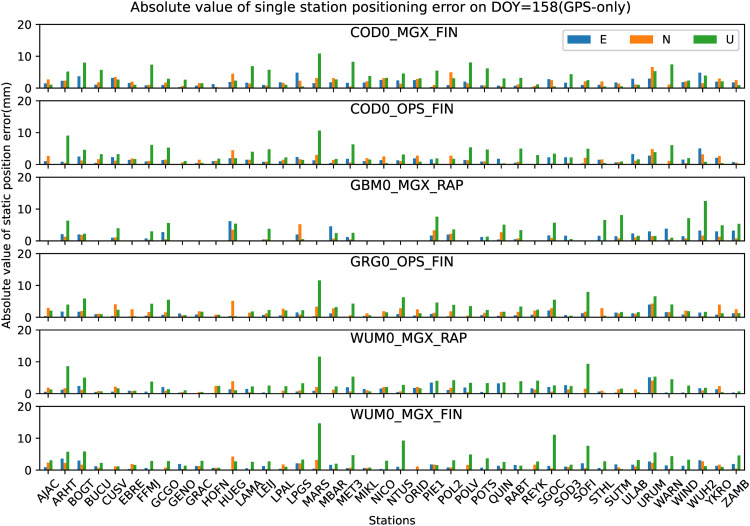
Positioning errors of the GPS-only static PPP-AR solutions 42 stations on DOY 158 in 2023 (Only GBM0_MGX_RAP product is available for 26 stations).

**Fig 3:**
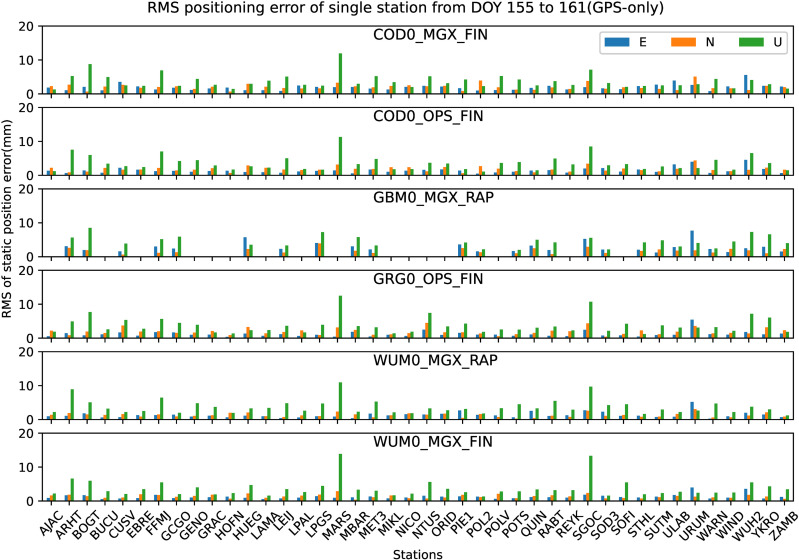
RMS values of GPS-only static PPP-AR positioning accuracy of each station with six PPP-AR products.

**Fig 4:**
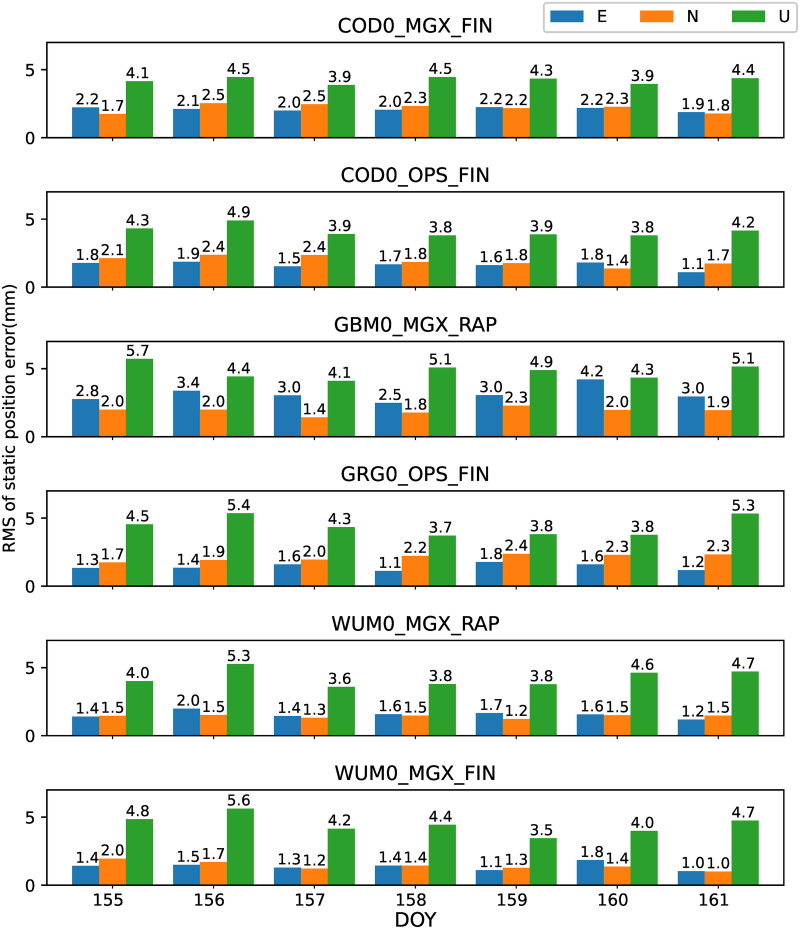
RMS time series of difference with IGS daily solutions among 7 days of GPS-only PPP-AR in east, north, and up components with six PPP-AR products.

**Fig 5:**
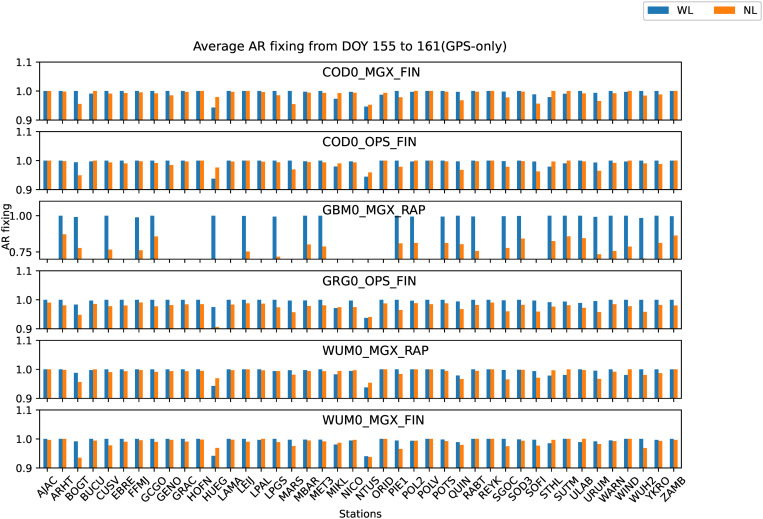
Average WL and NL AR fixing rate from DOY 155 to 161 of GPS-only for six PPP-AR products.

All the 42 JAVAD and/or LEICA receivers of stations shown in Fig 1 are used to analyze the preliminary performance of GPS-only static PPP-AR with six AC PPP-AR products. Among them, The GBM0_MGX_RAP GPS phase bias product is only available for 26 JAVAD receiver stations. Firstly, the positioning results of the GPS-only static PPP-AR with six AC PPP-AR products are shown in Fig 2, and the observations of 42 stations at DOY 158 in 2023. Secondly, take the root mean square (RMS) value for the 7-day positioning accuracy of each station, as shown in Fig 3. Next, the time series of static positioning RMS with six AC PPP-AR products are shown in Fig 4 and the RMS and AR fixing rate of all days with six AC PPP-AR products are listed in Table 5. Lastly, the average AR fixing rate from DOY 155–161 is shown in Fig 5.

**Table 5:** Mean of all daily RMS and AR fixing rate of GPS-only for six PPP-AR products.

Product type	RMS(mm)				AR fixing rate	
East	North	Up	3D	WL	NL
COD0_MGX_FIN	2.1	2.2	4.2	5.2	99.47%	98.89%
COD0_OPS_FIN	1.6	1.9	4.1	4.8	99.52%	98.99%
GBM0_MGX_RAP	3.1	1.9	4.8	6.0	99.70%	79.13%
GRG0_OPS_FIN	1.4	2.1	4.4	5.1	99.55%	97.50%
WUM0_MGX_RAP	1.5	1.4	4.3	4.8	99.37%	98.99%
WUM0_MGX_FIN	1.4	1.4	4.5	4.9	99.44%	98.80%

Fig 2 show that on DOY 158 in 2023, the positioning accuracy with COD0_MGX_FIN product in the east and north directions of each station is better than 6.6 mm, and the up direction is better than 10.9 mm; the positioning accuracy with COD0_OPS_FIN product in the east and north directions of each station is better than 5.1 mm, and the up direction is better than 10.7 mm; the positioning accuracy with GBM0_MGX_RAP product in the east and north directions of each station is better than 6.2 mm, and the up direction is better than 12.5 mm; the positioning accuracy with GRG0_OPS_FIN product in the east and north directions of each station is better than 5.2 mm, and the up direction is better than 11.6 mm; the positioning accuracy with WUM0_MGX_RAP product in the east and north directions of each station is better than 5.1 mm, and the up direction is better than 11.6 mm; the positioning accuracy with WUM0_MGX_FIN product in the east and north directions of each station is better than 4.3 mm, and the up direction is better than 14.7 mm.

From Fig 3, it can be concluded that the results with different AC PPP-AR products were overall consistent with each other. Fig 3 show that RMS values from DOY 158–161 in 2023, the positioning accuracy with COD0_MGX_FIN product in the east and north directions of each station is better than 5.6 mm, and the up direction is better than 12.0 mm; the positioning accuracy with COD0_OPS_FIN product in the east and north directions of each station is better than 4.6 mm, and the up direction is better than 11.4 mm; the positioning accuracy with GBM0_MGX_RAP product in the east and north directions of each station is better than 7.7 mm and 4.0 mm respectively, and the up direction is better than 8.5 mm; the positioning accuracy with GRG0_OPS_FIN product in the east and north directions of each station is better than 5.5 mm, and the up direction is better than 12.6 mm; the positioning accuracy with WUM0_MGX_RAP product in the east and north directions of each station is better than 5.3 mm, and the up direction is better than 11.0 mm; the positioning accuracy with WUM0_MGX_FIN product in the east and north directions of each station is better than 4.0 mm, and the up direction is better than 13.9 mm.

Figs 2 and 3 show that the positioning accuracy varied significantly between stations.

Fig 4 shows that the positioning performance is stable with six AC PPP-AR products over 7 days. The maximum daily RMS differences with COD0_MGX_FIN product 7 days are 0.3 mm, 0.8 mm, and 0.6 mm for the east, north, and up components, respectively. The maximum daily RMS differences with COD0_OPS_FIN product 7 days are 0.8 mm, 1.0 mm, and 1.1 mm, respectively. The maximum daily RMS differences with GBM0_MGX_RAP product 7 days are 1.4 mm, 0.9 mm, and 1.1 mm, respectively. The maximum daily RMS differences with GRG0_OPS_FIN product 7 days are 0.7 mm, 0.6 mm, and 1.4 mm, respectively. The maximum daily RMS differences with WUM0_MGX_RAP product 7 days are 0.8 mm, 0.3 mm, and 1.5 mm, respectively. The maximum daily RMS differences with WUM0_MGX_FIN product 7 days are 0.8 mm, 1.0 mm, and 2.1 mm, respectively. In terms of stability, Fig 4 shows that the COD0_MGX_FIN product performs best, COD0_OPS_FIN GRG0_OPS_FIN and WUM0_MGX_RAP products perform well in terms of stability, GBM0_MGX_RAP product is average. WUM0_MGX_FIN product is the worst performing. In terms of stability, Fig 4 shows that the COD0_MGX_FIN product performs best.

The statistical results in Table 5 show that the RMS solutions with different AC PPP-AR products. Overall, COD0_OPS_FIN, WUM0_MGX_RAP, and WUM0_MGX_FIN product performs best in east, north, and up components, and the positioning RMS in three-dimensional (3D) direction is better than 5 mm. COD0_MGX_FIN and GRG0_OPS_FIN products take second place, and the positioning accuracy in the 3D direction is slightly greater than 5 mm. GBM0_MGX_RAP product is relatively poor, and positioning RMS is 3.1 mm in east components, but the RMS values of the north and up components are comparable to the other five products, and the positioning accuracy in the 3D direction has reached 6 mm.

Fig 5 shows the mean AR fixing rate of GPS-only WL and NL for each observation station from DOY 155–161 with six PPP-AR products, AR WL and NL fixing rate is defined as follows:


$$r_{WL}=\frac{N_{WL}}{N_{all}}$$ (9)

$$r_{NL}=\frac{N_{NL}}{N_{WL}}$$ (10)

where $r_{WL}$ represents the WL fixing rate; $N_{all}$ represents the number of independent all ambiguities; $N_{WL}$ represents the number of independent WL fixed ambiguities, $r_{NL}$ represents the NL fixing rate; $N_{NL}$ represents the number of independent NL fixed ambiguities.

Fig 5 shows AR NL fixing rate of the GBM0_MGX_RAP product is relatively poor, but the WL fixing rate is similar to the other five products. The other five products are relatively similar in the WL and NL fixing rates, and the WL and NL fixing rates of all stations exhibit the same trend. As seen in Table 5, no significant difference was found for WL fixing rates of GPS-only for six PPP-AR products, WL fixing rate of all products is over 99%, nevertheless, NL fixing rates of GBM0_MGX_RAP products are much lower than the other five products. Compared to other products, the NL fixing rate of the GBM0_MGX_RAP product is 20% lower. The NL fixing rate of COD0_OPS_FIN and WUM0_MGX_RAP products has reached 98.99%. NL fixing rate of COD0_MGX_FIN, GRG0_OPS_FIN, and WUM0_MGX_FIN products are not significantly different with COD0_OPS_FIN and WUM0_MGX_RAP products. Except for the GBM0_MGX_RAP product, they all achieved a relatively high NL fixing rate.

### 4.2. PPP-AR performance of Galileo-only

**Fig 6:**
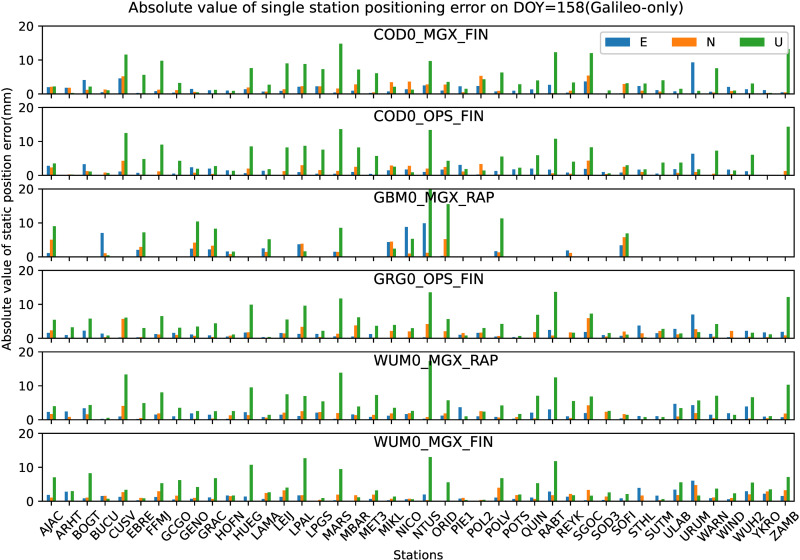
Positioning errors of the Galileo-only static PPP-AR solutions 42 stations on DOY 158 in 2023 (Only GBM0_MGX_RAP products is available for 16 stations).

**Fig 7:**
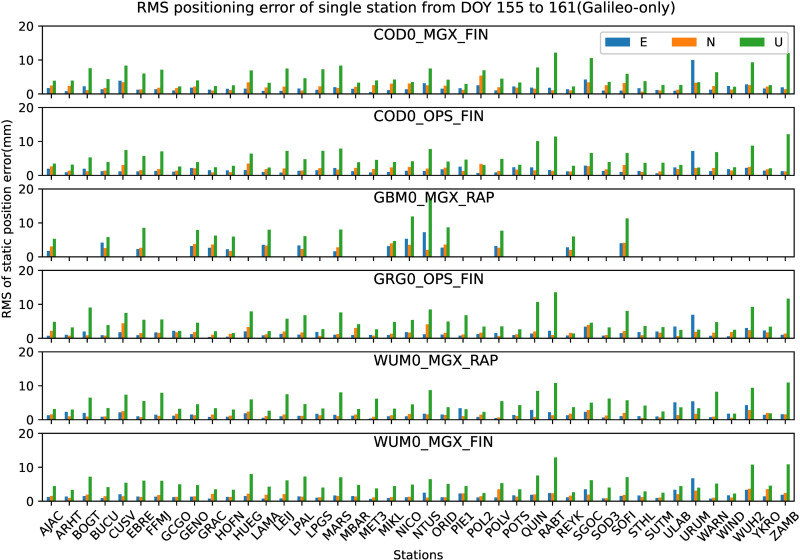
RMS values of Galileo-only static PPP-AR positioning accuracy of each station with six PPP-AR products.

**Fig 8:**
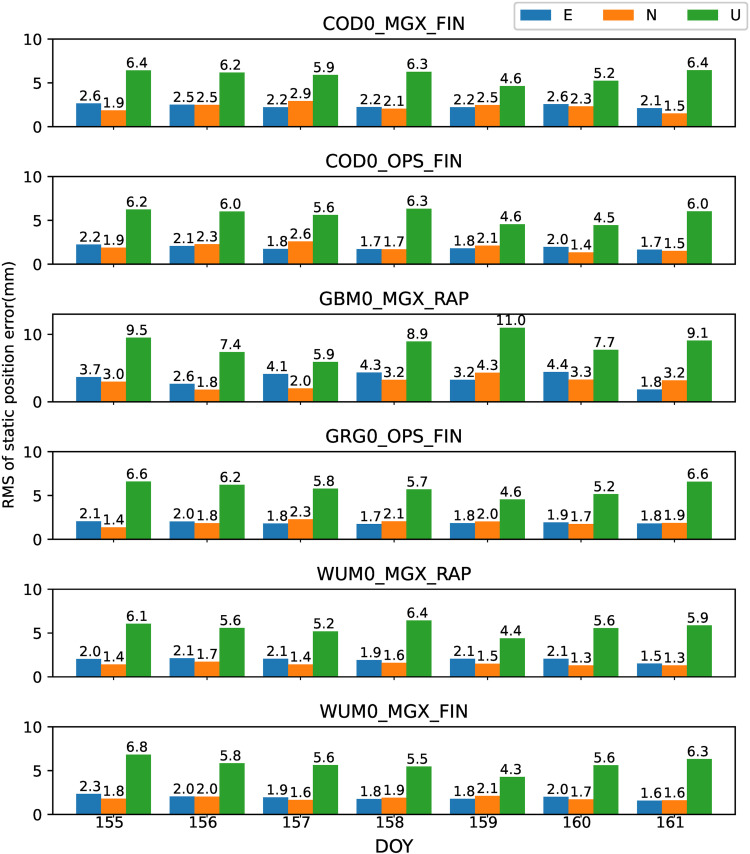
RMS time series of difference with IGS daily solutions among 7 days of Galileo-only PPP-AR in east, north, and up components with six PPP-AR products.

**Fig 9:**
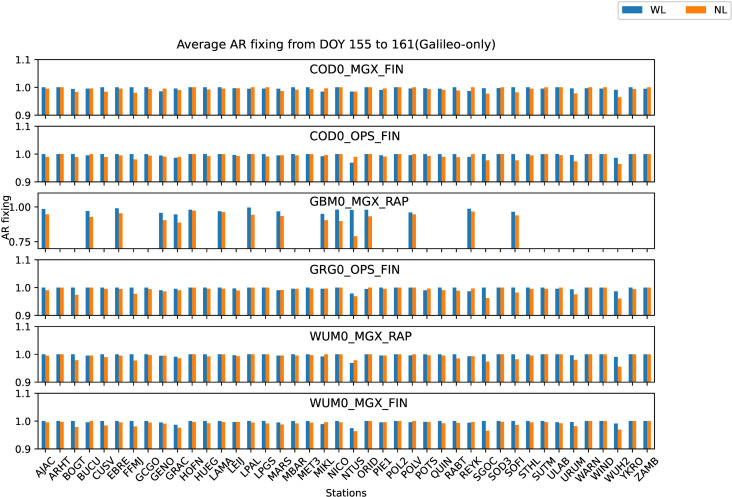
Average WL and NL AR fixing rate from DOY 155 to 161 of Galileo-only for six PPP-AR products.

Similar to the analysis of GPS-only in section 4.1, all the 42 JAVAD and/or LEICA receivers of stations shown in Fig 1 are used to analyze the preliminary performance of Galileo-only static PPP-AR with six AC PPP-AR products. The GBM0_MGX_RAP Galileo phase bias product is only available for 16 LEICA receiver stations. Firstly, the positioning results of the Galileo-only static PPP-AR with six AC PPP-AR products are shown in Fig 6, and the observations of 42 stations at DOY 158 in 2023. Secondly, take the root mean square (RMS) value for the 7-day positioning accuracy of each station, as shown in Fig 7. Next, the time series of static positioning RMS with six AC PPP-AR products are shown in Fig 8 and the RMS and AR fixing rate of all days with six AC PPP-AR products are listed in Table 6. Lastly, the average AR fixing rate from DOY 155–161 is shown in Fig 9.

**Table 6:** Mean of all daily RMS and AR fixing rate of Galileo-only for six PPP-AR products.

Product type	RMS(mm)				Fixing rate	
**East**	**North**	**Up**	**3D**	**WL**	**NL**
**COD0_MGX_FIN**	2.4	2.2	5.9	6.7	99.64%	99.31%
**COD0_OPS_FIN**	1.9	1.9	5.6	6.2	99.75%	99.34%
**GBM0_MGX_RAP**	3.5	3.0	8.5	9.7	97.24%	92.56%
**GRG0_OPS_FIN**	1.9	1.9	5.8	6.4	99.74%	99.20%
**WUM0_MGX_RAP**	2.0	1.5	5.6	6.1	99.78%	99.32%
**WUM0_MGX_FIN**	1.9	1.8	5.7	6.3	99.75%	99.15%

Figs 6 and 7 show that the Galileo-only positioning accuracy varied significantly between stations, which are the same result as GPS-only.

Fig 6 show that on DOY 158 in 2023, the positioning accuracy with COD0_MGX_FIN product in the east directions of each station is better than 9.3 mm, the north direction is better than 5.4 mm and the up direction is better than 14.8 mm; the positioning accuracy with COD0_OPS_FIN product in the east directions of each station is better than 6.4 mm, the north direction is better than4.3 mm and the up direction is better than 14.3 mm; the positioning accuracy with GBM0_MGX_RAP product in the east directions of each station is better than 9.9 mm, the north direction is better than 5.8 mm and the up direction is better than 20.3 mm; the positioning accuracy with GRG0_OPS_FIN product in the east directions of each station is better than 7.0 mm, the north direction is better than 5.9 mm and the up direction is better than 13.7 mm; the positioning accuracy with WUM0_MGX_RAP product in the east and north directions of each station is better than 4.7 mm, and the up direction is better than 17.3 mm; the positioning accuracy with WUM0_MGX_FIN product in the east directions of each station is better than 6.1 mm, the north direction is better than 4.7 mm and the up direction is better than 13.0 mm.

Fig 7 show that RMS values of all stations from DOY 158–161 in 2023, the positioning accuracy with COD0_MGX_FIN product in the east and north directions of each station is better than 10.0 mm and 5.4 mm respectively, and the up direction is better than 12.1 mm; the positioning accuracy with COD0_OPS_FIN product in the east and north directions of each station is better than 7.2 mm and 3.5 mm respectively, and the up direction is better than 12.1 mm; the positioning accuracy with GBM0_MGX_RAP product in the east and north directions of each station is better than 7.2 mm and 4.1 mm respectively, and the up direction is better than 17.2 mm; the positioning accuracy with GRG0_OPS_FIN product in the east and north directions of each station is better than 6.9 mm and 4.4 mm respectively, and the up direction is better than 13.5 mm; the positioning accuracy with WUM0_MGX_RAP product in the east and north directions of each station is better than 5.4 mm and 2.9 mm respectively, and the up direction is better than 11.0 mm; the positioning accuracy with WUM0_MGX_FIN product in the east and north directions of each station is better than 6.7 mm and 3.7 mm respectively, and the up direction is better than 12.9 mm. From Fig 7, it can be concluded that the results with different AC PPP-AR products were overall consistent with each other. The maximum positioning accuracy of the WUM0_MGX_RAP product is the best; the GBM0_MGX_RAP product has the worst performance; the other four products are in the middle, and the difference is not significant.

Fig 8 shows that the positioning performance is stable with six AC PPP-AR products over 7 days. The maximum daily RMS differences with COD0_MGX_FIN product 7 days are 0.5 mm, 1.0 mm, and 1.8 mm for the east, north, and up components, respectively. The maximum daily RMS differences with COD0_OPS_FIN product 7 days are 0.5 mm, 0.9 mm, and 1.8 mm, respectively. The maximum daily RMS differences with GBM0_MGX_RAP product 7 days are 2.6 mm, 1.5 mm, and 3.6 mm, respectively. The maximum daily RMS differences with GRG0_OPS_FIN product 7 days are 0.4 mm, 0.9 mm, and 2.0 mm, respectively. The maximum daily RMS differences with WUM0_MGX_RAP product 7 days are 0.6 mm, 0.4 mm, and 2.0 mm, respectively. The maximum daily RMS differences with WUM0_MGX_FIN product 7 days are 1.3 mm, 0.5 mm, and 2.5 mm, respectively. In terms of stability, Fig 7 shows that COD0_MGX_FIN, COD0_OPS_FIN, GRG0_OPS_FIN, and WUM0_MGX_RAP product performs best, WUM0_MGX_FIN product performance takes second place, GBM0_MGX_RAP product is the worst performing in terms of stability.

The Galileo-only statistic results in Table 6 show that the RMS solutions with different AC PPP-AR products. Overall, COD0_OPS_FIN, GRG0_OPS_FIN, WUM0_MGX_RAP, and WUM0_MGX_FIN products perform best in east, north, and up components, and the positioning RMS in three-dimensional (3D) direction is better than 6.5 mm. COD0_MGX_FIN product takes second place, and the positioning accuracy in the 3D direction accuracy does not exceed 7 mm. GBM0_MGX_RAP product is relatively poor, and 3D positioning RMS is approximately 10 mm, the RMS values of the east, north, and up components are inferior to the other five products.

Fig 9 shows the mean AR fixing rate of Galileo-only WL and NL for each observation station from DOY 155–161 with six PPP-AR products, AR WL and NL fixing rate is defined as Section 4.1. Fig 9 shows partial stations AR NL fixing rate of the GBM0_MGX_RAP product is relatively poor, but the WL fixing rate is not much different from the other five products. The other five products are relatively similar in the WL and NL fixing rates, and the WL and NL fixing rates of all stations also exhibit the same trend as GPS-only. As seen in Table 6, the WL and NL fixing rates of COD0_MGX_FIN, COD0_OPS_FIN, GRG0_OPS_FIN, WUM0_MGX_RAP, and WUM0_MGX_FIN products have all exceeded 99%, they all achieved a relatively high fixing rate. The WL fixing rate of the GBM0_MGX_RAP product is 97.24%, about 2.5% lower than other products. The NL fixing rate of the GBM0_MGX_RAP product is 92.56%, about 7% lower than other products.

It is recommended to regularly display the kinematic positioning errors of some specific stations with various IGS/MGEX final products on the IGS official website as another measure of the quality of satellite orbit, clock products, and code/phase bias. Similar results were also reported by [33] and [52].

## 5. Discussion

This article selects 42 IGS/MGEX tracking stations worldwide and uses 6 products released by 4 ACs for PPP-AR calculation. The positioning results of the stations are closely related to the quality of PPP-AR products. The selected stations, algorithms, and calculation strategies used by each AC in generating network solution precision products are different, resulting in differences in the generated products. This directly affects the performance of users when using corresponding precision products for PPP-AR in practical applications.

Tables 7–11 show some strategies for different products, and different ACs use different GNSS data processing software platforms, such as Bernese software developed by CODE, GFZ’s EPOS software, CNES’s Gins software, and Wuhan university’s independently developed PANDA software. The detailed analysis strategy summary can be developed through https://files.igs.org/pub/center/analysis and obtain ACN files for different products. The CODE was the first to release OSB products, utilizing the common clock (CC) model, which is equivalent to the IRC model, to generate the bias products. The GFZ WL UPDs and re-estimated clocks, combined with IF UPDs from the CNES, are based on the IRC approach. Starting from September 2021, CNES has also started to release OSB products. The CNES/CLS AC is performing PPP-AR using the zero-difference ambiguity fixing approach also known as IRC model. Like CODE, WUM rapid utilized the phase clock/biases model to generate products that are mathematically equivalent to the IRC model. The WUM final uses UPD model.

**Table 7:** Partial data processing strategy of the CODE products.

Software Used	Bernese GNSS Software Version 5.5, developed at AIUB
Measurement models	
Basic Observable	GPS/GLONASS/Galileo (/BDS/QZSS) carrier phase; code only used for receiver clock synchronization and MW ambiguity resolution. Elevation angle cutoff: 3 degrees Sampling rate: 3 minutes Weighting: 6 mm for double-differenced ionosphere-free phase observations at zenith; elevation-dependent weighting function 1/cos(z)**2
Modeled observables	Double differences, ionosphere-free linear combination observables of two frequencies
Estimated parameters (a priori values and constraints)	
Adjustment	Weighted least-squares algorithms
Station coordinates	All station coordinates are adjusted with minimum coordinates constraints,
Troposphere	Zenith delay: estimated for each station in intervals of 2 hours. Loose relative constraints of 5 m are applied. Piece-wise, linear parameterizations, allowing for connection of the parameters at day boundaries. Zenith delay epochs: every two hours starting at midnight Mapping function: wet VMF3 for final; Gradients: pairs of horizontal delay gradient parameters are estimated in N-S and E-W direction for each station in intervals of 24 hours. Loose relative constraints of 5 m are applied. Piece-wise, linear parameterizations, allowing for connection of the parameters at day boundaries.
Ionospheric correction	Not estimated in ionosphere-free analyses One scaling factor for 2nd and 3rd order terms and ray bending is setup to switch the components on or off on normal equation level. The products are generated with considering all three correction components.
Ambiguities	Ambiguities are resolved in a baseline-by-baseline mode performing the following steps: Melbourne-Wuebbena approach (< 6000 km); Quasi-Ionosphere-Free (QIF) approach (< 2000 km); Phase-based WL/NL method (< 200 km); Direct L1/L2 method, also for GLONASS (< 20 km); GNSS-derived global ionosphere map information is used to support the code-less methods.

**Table 8:** Partial Data processing strategy of the GBM0_MGX_RAP product.

Software Used	EPOS.P8 developed at GFZ
Measurement models	
Basic Observable	carrier phases and pseudo-ranges elevation angle cutoff: 3 degrees sampling rate: 5 minutes weighting: elev.dep. weighting with unit weight until 30 deg, 1/2sin(e) below
Modeled observables	undifferenced observables, corrected for 1st order effect by forming ionosphere-free linear combination
Estimated parameters (apriori values and constraints)	
Adjustment	least-square adjustment
Station coordinates	station coordinates constrained to IGb20.snx with post sesmic correction
Troposphere	zenith delay: zenith delay parameters for each station with 1 hour intervals (random walk) mapping function: wet Vienna Mapping Functions (VMF) zenith delay epochs: each integer hour gradients: north and east horizontal delay are estimated for each station in daily intervals
Ionospheric corrections	not estimated
Ambiguities	double-difference ambiguities are fixed according to Ge et al.(2005); un-difference ambiguities are fixed according to Deng et al.(2022)

**Table 9:** Partial Data processing strategy of the GRG0_OPS_FIN product.

Software Used	GINS Software developed by CNES for orbit computation and NEQ building
Measurement models	
Basic Observable	Undifferenced Carrier phase + code ionosperic free for ambiguity fixing and unfixed passes Elevation angle cutoff: 8 degrees from wwww = 1997 Sampling rate: 5 minutes for orbit/sinex 30 secs for clock densification Weighting: Constellation dependent 3.5 mm/ 60 cm for GPS phase/code 5.2 mm/ 60 cm for GALILEO phase/code Use of specific elevation weighting law (phase/range) sigma = sig0/ (a + (1-a) sin(elev))
Modeled observables	Undifferenced, ionosphere-free linear combination carrier phase (and code for unfixed passes)
Troposphere a priori model	Meteo data input: Dry and wet a priori zenithal delays interpolated from ECMWF 6-hour grids Since week 2238: Use of VMF1/VMF1 Estimated parameters: 12 zenith delay/day using piecewise linear, continuous model + one tropospheric gradient per station in North and East direction Loose constraints: only for undetermined parameters Mapping function: VMF1
Ionosphere	Not modeled (first-order effect eliminated by forming the ionosphere-free linear combination of L1 and L2) 2nd order effects applied after week 1759 computed Hernandez-Parajes, 2007 (TEC from IGS/IGR iono grids)
Estimated parameters (a priori values and constraints)	
Adjustment	Weighted least-squares algorithms
Station coordinates	All station coordinates are adjusted, relative to the a priori values from ITRF2005; minimal constrains are applied to all coordinates Use of IGb20 since week 2238
Troposphere	Zenith delay parameters are estimated for each station using a piecewise linear continuous model with 12 parameters/day
Ionospheric correction	Not estimated in ionosphere-free analyses
Ambiguities	One bias per pass for remaining unfixed ambiguities (more than 95% are fixed)

**Table 10:** Partial Data processing strategy of the WUM_MGX_RAP product.

Software Used	PANDA (Position And Navigation Data Analyst), developed at WHU
Measurement models	
Basic Observable	carrier phases and pseudo-ranges Elevation angle cutoff: 7 degrees for all systems Sampling rate: 300 seconds for POD, 30 seconds for PPP and clock densification. Weighting: 0.01 cycles and 0.5 m for GPS/Galileo raw phase and code observations at zenith; elev.dep. weighting with unit weight until 30 deg, 1/2sin(e) below
Modeled observables	undifferenced observables, corrected for 1st order effect by forming ionosphere-free linear combination
Estimated parameters (apriori values and constraints)	
Adjustment	Weighted least-squares algorithm
Station coordinates	station coordinates constrained to igsyyPwwww.snx for POD, PPP and clock densification.
Troposphere	Zenith delay: estimated for each station in intervals of 1 hours. Loose relative constraints of 0.1 m are applied. Piece-wise constant parametrization. Zenith delay epochs: every one hours starting at midnight Mapping function: VMF3 gradients: north and east horizontal delay are estimated for each station in daily intervals
Ionospheric correction	Not estimated in ionosphere-free analysis
Ambiguities	ambiguities are fixed according to [42,61] for GPS/Galileo/BDS

**Table 11:** Partial Data processing strategy of the WUM_MGX_FIN product.

Software Used	PANDA (Position And Navigation Data Analyst), developed at WHU
Measurement models	
Basic Observable	carrier phases and pseudo-ranges Elevation angle cutoff: 7 degrees for GPS,10 degree for Galileo, Sampling rate: 300 second for POD of GPS. 30 second for high-rate clock generation of GPS and GLONASS as well as PPP and POD of Galileo, BeiDou, and QZSS Weighting: 2 cm and 2 m for raw phase and code observations at zenith; elev.dep. weighting with unit weight until 30 deg, 1/2sin(e) below
Modeled observables	undifferenced observables, corrected for 1st order effect by forming ionosphere-free linear combination
Estimated parameters (a priori values and constraints)	
Adjustment	Weighted least-squares algorithms
Station coordinates	station coordinates constrained to IGb14.snx for GPS and GLONASS POD; All are fixed as PPP estimation for Galieo/BeiDou/QZSS POD.
Troposphere	Zenith delay: estimated for each station in intervals of 2 hours. Loose relative constraints of 1 m are applied. Piece-wise constant parametrization. Zenith delay epochs: every two hours starting at midnight Mapping function: wet VMF1 gradients: north and east horizontal delay are estimated for each station in daily intervals
Ionospheric correction	Not estimated in ionosphere-free analyses
Ambiguities	ambiguities are fixed according to [61] for GPS/Galieo/BeiDou

PPP-AR users must undergo strict error model correction during the calculation process, while also striving to maintain consistency with the calculation strategy and model provided by the phase bias product. Otherwise, model inconsistency can easily lead to a lower fixed rate of PPP ambiguity, or even errors in ambiguity fixation, which will undoubtedly have a serious impact on the final PPP results of users, and even result in PPP-AR results being inferior to PPP floating-point solutions. The product positioning accuracy of WUM and CODE ACs is relatively high. However, the positioning accuracy of GBM product analysis center is poor. The main reason is that the PPP-AR program used in WUM products and software model has good self consistency, and the model used in the CODE AC is also the most consistent with the software model, so it is slightly better than GBM’s. This phenomenon can also be observed from the NL fixed rate of ambiguity.

## 6. Conclusions

The GPS-only and Galileo-only positioning performance of PPP-AR precise products in static mode was evaluated and comprehensively analyzed in this article. The contribution of six PPP-AR precise products (i.e., COD0_MGX_FIN, COD0_OPS_FIN, GBM0_MGX_RAP, GRG0_OPS_FIN, WUM0_MGX_RAP, and WUM0_MGX_FIN) on GPS-only, and Galileo-only positioning performance with PPP-AR has been comprehensively investigated and evaluated in static mode based on 7-days observations (DOY 155–161 in 2023) of 42 MGEX stations capable of tracking GPS and Galileo signals. It should be noted that during the PPP-AR processing with the various AC products, the selected software (i.e., PRIDE PPP-AR software) and processing settings (i.e., dual-frequency ionosphere-free combined observables) are the same; hence, the only variable is a complete set of precise products including code/phase bias, satellite orbit, clock, ERP, and satellite attitude quaternion.

For GPS-only PPP-AR, the positioning performance in terms of positioning accuracy derived from static PPP solutions with the five AC products (i.e., COD0_MGX_FIN, COD0_OPS_FIN, GRG0_OPS_FIN, WUM0_MGX_RAP, and WUM0_MGX_FIN) demonstrated a good agreement with each other, while those with the one AC products (i.e., GBM0_MGX_RAP) showed much worse performance. Although the differences in positioning accuracy are very small, the PPP-AR performance with COD0_OPS_FIN, WUM0_MGX_RAP, and WUM0_MGX_FIN products provided by CODE and WHU rank the first, COD0_MGX_FIN, and GRG0_OPS_FIN products provided by CODE and CNES ranked second, while those with the GBM0_MGX_RAP product provided by GFZ showed worse performance. Nevertheless, with different AC products, obvious differences in positioning errors for some specific stations can be observed. The RMS of static positioning using five products (i.e., COD0_MGX_FIN, COD0_OPS_FIN, GRG0_OPS_FIN, WUM0_MGX_RAP, and WUM0_MGX_FIN) reached about 2 mm, 2 mm, 4 mm, and 5 mm in east, north, up and 3D components, respectively, GBM0_MGX_RAP products reached about 3 mm, 2 mm, 4 mm, and 6 mm in east, north, up and 3D components, respectively. The WL fixing rate of GPS-only showed a good agreement with each other, and all products have over 99%. But compared to other products, the NL fixing rate of five products (i.e., COD0_MGX_FIN, COD0_OPS_FIN, GRG0_OPS_FIN, WUM0_MGX_RAP, and WUM0_MGX_FIN) reached about 98%, and GBM0_MGX_RAP products reached about 79%, GBM0_MGX_RAP product has a fixing rate approximately 20% lower than other five products.

For Galileo-only PPP-AR, the positioning performance in terms of positioning accuracy derived from static PPP solutions with the five AC products (i.e., COD0_MGX_FIN, COD0_OPS_FIN, GRG0_OPS_FIN, WUM0_MGX_RAP, and WUM0_MGX_FIN) demonstrated a good agreement with each other, while those with the one AC products (i.e., GBM0_MGX_RAP) showed much worse performance. Although the differences in positioning accuracy are very small, the PPP-AR performance with COD0_OPS_FIN, GRG0_OPS_FIN, WUM0_MGX_RAP, and WUM0_MGX_FIN products provided by CODE, CNES and WHU rank first, COD0_MGX_FIN product provided by CODE ranked second, while those with the GBM0_MGX_RAP product provided by GFZ showed worse performance. Different products exhibit almost identical positioning performance under GPS-only and Galileo–only. The RMS of static positioning using four products (i.e., COD0_OPS_FIN, GRG0_OPS_FIN, WUM0_MGX_RAP, and WUM0_MGX_FIN) reached about 2 mm, 1.9 mm, 5.8 mm, and 6.4 mm in east, north, up, and 3D components, respectively, COD0_MGX_FIN product reached 2.4 mm, 2.2 mm, 5.9 mm, and 6.7 mm in east, north, up, and, 3D components, respectively. GBM0_MGX_RAP product reached about 3.5 mm, 3.0 mm, 8.5 mm, and 9.7 mm in east, north, up, and 3D components, respectively. The WL and NL fixing rate of Galileo-only showed five products (i.e., COD0_MGX_FIN, COD0_OPS_FIN, GRG0_OPS_FIN, WUM0_MGX_RAP, and WUM0_MGX_FIN) have all exceeded 99%, they all achieved a relatively high fixing rate. The WL and NL fixing rates of the GBM0_MGX_RAP product are 97.24% and 92.56%, respectively. Compared to other products, WL and NL fixing rates are about 2.5% and 7% respectively. The above analysis indicates that, positioning accuracy is related to AR fixing rate. When using the same PPP-AR product, GPS-only has higher positioning performance than Galileo-only.

## References

[1] Zumberge JF, Heflin MB, Jefferson DC, Watkins MM, Webb FH. Precise point positioning for the efficient and robust analysis of GPS data from large networks. J Geophys Res. 1997;102(B3):5005–17. doi: 10.1029/96jb03860

[2] Geng J, Teferle FN, Meng X, Dodson AH. Towards PPP-RTK: Ambiguity resolution in real-time precise point positioning. Ad Space Res. 2011;47(10):1664–73. doi: 10.1016/j.asr.2010.03.030

[3] Geng J, Pan Y, Li X, Guo J, Liu J, Chen X, et al. Noise Characteristics of High‐Rate Multi‐GNSS for Subdaily Crustal Deformation Monitoring. JGR Solid Earth. 2018;123(2):1987–2002. doi: 10.1002/2018jb015527

[4] Su K, Jin S, Ge Y. Rapid displacement determination with a stand-alone multi-GNSS receiver: GPS, Beidou, GLONASS, and Galileo. GPS Solut. 2019;23(2). doi: 10.1007/s10291-019-0840-4

[5] Yuan Y, Zhang K, Rohm W, Choy S, Norman R, Wang C. Real‐time retrieval of precipitable water vapor from GPS precise point positioning. JGR Atmospheres. 2014;119(16):10044–57. doi: 10.1002/2014jd021486

[6] Li X, Dick G, Lu C, Ge M, Nilsson T, Ning T, et al. Multi-GNSS Meteorology: Real-Time Retrieving of Atmospheric Water Vapor From BeiDou, Galileo, GLONASS, and GPS Observations. IEEE Trans Geosci Remote Sensing. 2015;53(12):6385–93. doi: 10.1109/tgrs.2015.2438395

[7] Pan L, Zhang X, Guo F, Liu J. GPS inter-frequency clock bias estimation for both uncombined and ionospheric-free combined triple-frequency precise point positioning. J Geod. 2018;93(4):473–87. doi: 10.1007/s00190-018-1176-5

[8] Ge M, Gendt G, Rothacher M, Shi C, Liu J. Resolution of GPS carrier-phase ambiguities in Precise Point Positioning (PPP) with daily observations. J Geod. 2007;82(7):389–99. doi: 10.1007/s00190-007-0187-4

[9] Håkansson M, Jensen ABO, Horemuz M, Hedling G. Review of code and phase biases in multi-GNSS positioning. GPS Solut. 2016;21(3):849–60. doi: 10.1007/s10291-016-0572-7

[10] Ogutcu S. Performance analysis of ambiguity resolution on PPP and relative positioning techniques: consideration of satellite geometry. International Journal of Engineering and Geosciences. 2020;5(2):73–93. doi: 10.26833/ijeg.580027

[11] Geng J, Meng X, Dodson AH, Ge M, Teferle FN. Rapid re-convergences to ambiguity-fixed solutions in precise point positioning. J Geod. 2010;84(12):705–14. doi: 10.1007/s00190-010-0404-4

[12] Atiz OF, Ogutcu S, Alcay S, Li P, Bugdayci I. Performance investigation of LAMBDA and bootstrapping methods for PPP narrow-lane ambiguity resolution. Geo-spatial Information Science. 2021;24(4):604–14. doi: 10.1080/10095020.2021.1942236

[13] Banville S, Hassen E, Lamothe P, Farinaccio J, Donahue B, Mireault Y, et al. Enabling ambiguity resolution in CSRS‐PPP. Navigation. 2021;68(2):433–51. doi: 10.1002/navi.423

[14] Yue C, Dang Y, Xue S, Wang H, Gu S, Xu C. A New Optimal Subset Selection Method of Partial Ambiguity Resolution for Precise Point Positioning. Remote Sensing. 2022;14(19):4819. doi: 10.3390/rs14194819

[15] Zhao L, Blunt P, Yang L, Ince S. Performance Analysis of Real-Time GPS/Galileo Precise Point Positioning Integrated with Inertial Navigation System. Sensors (Basel). 2023;23(5):2396. doi: 10.3390/s23052396 36904600 PMC10007143

[16] Yue C, Dang Y, Gu S, Wang H, Zhang J. Optimization of undifferenced and uncombined PPP stochastic model based on covariance component estimation. GPS Solut. 2022;26(4). doi: 10.1007/s10291-022-01310-7

[17] Plata I, Humez P, Wilson L, Nightingale M, McClain C, Mayer B. Distribution, sources, and fate of nitrate in groundwater in agricultural areas of Southern Alberta, Canada. Biogeochemistry. 2025;168(1):18. doi: 10.1007/s10533-025-01209-8 39925730 PMC11799072

[18] Qu L, Du Y, Wang H, Jiang W, Wang L. Multi-constellation and multi-frequency precise point positioning with fast ambiguity resolution on a global scale. Measurement. 2023;211:112642. doi: 10.1016/j.measurement.2023.112642

[19] Wang N, Yuan Y, Li Z, Montenbruck O, Tan B. Determination of differential code biases with multi-GNSS observations. J Geod. 2015;90(3):209–28. doi: 10.1007/s00190-015-0867-4

[20] Li M, Yuan Y. Estimation and Analysis of BDS2 and BDS3 Differential Code Biases and Global Ionospheric Maps Using BDS Observations. Remote Sensing. 2021;13(3):370. doi: 10.3390/rs13030370

[21] Jin R, Jin S, Feng G. M_DCB: Matlab code for estimating GNSS satellite and receiver differential code biases. GPS Solut. 2012;16(4):541–8. doi: 10.1007/s10291-012-0279-3

[22] Yue C, Wang H, Xu C, Dang Y, Gu S, Chen H. M_IFCB: a MATLAB-based software for multi‑GNSS inter‑frequency clock bias estimation and forecast. GPS Solut. 2024;28(4). doi: 10.1007/s10291-024-01687-7

[23] Liu T, Zhang B, Yuan Y, Li Z, Wang N. Multi-GNSS triple-frequency differential code bias (DCB) determination with precise point positioning (PPP). J Geod. 2018;93(5):765–84. doi: 10.1007/s00190-018-1194-3

[24] Li X, Zheng H, Li X, Yuan Y, Wu J, Han X. Open-source software for multi-GNSS inter-frequency clock bias estimation. GPS Solut. 2023;27(2). doi: 10.1007/s10291-023-01398-5

[25] Geng J, Shi C, Ge M, Dodson AH, Lou Y, Zhao Q, et al. Improving the estimation of fractional-cycle biases for ambiguity resolution in precise point positioning. J Geod. 2011;86(8):579–89. doi: 10.1007/s00190-011-0537-0

[26] Li X, Zhang X. Improving the Estimation of Uncalibrated Fractional Phase Offsets for PPP Ambiguity Resolution. J Navigation. 2012;65(3):513–29. doi: 10.1017/s0373463312000112

[27] Laurichesse D, Mercier F, Berthias J-P, Broca P, Cerri L. Integer Ambiguity Resolution on Undifferenced GPS Phase Measurements and Its Application to PPP and Satellite Precise Orbit Determination. Navigation. 2009;56(2):135–49. doi: 10.1002/j.2161-4296.2009.tb01750.x

[28] Guo F, Zhang X, Wang J. Timing group delay and differential code bias corrections for BeiDou positioning. J Geod. 2015;89(5):427–45. doi: 10.1007/s00190-015-0788-2

[29] Collins P, Bisnath S, Lahaye F, Héroux P. Undifferenced GPS Ambiguity Resolution Using the Decoupled Clock Model and Ambiguity Datum Fixing. Navigation. 2010;57(2):123–35. doi: 10.1002/j.2161-4296.2010.tb01772.x

[30] Lv J, Gao Z, Peng J. Modeling and Assessment of BDS/GPS Triple-Frequency Precise Point Positioning. In: Sun J, Yang C, Xie J, editors. China Satellite Navigation Conference (CSNC) 2020 Proceedings: Volume II Singapore: Springer Nature Singapore; 2020. pp. 201–210. doi: 10.1007/978-981-15-3711-0_18

[31] Chen C, Xiao G, Chang G, Xu T, Yang L. Assessment of GPS/Galileo/BDS Precise Point Positioning with Ambiguity Resolution Using Products from Different Analysis Centers. Remote Sensing. 2021;13(16):3266. 10.3390/rs13163266

[32] Geng J, Meng X, Dodson AH, Teferle FN. Integer ambiguity resolution in precise point positioning: method comparison. J Geod. 2010;84(9):569–81. doi: 10.1007/s00190-010-0399-x

[33] Glaner M, Weber R. PPP with integer ambiguity resolution for GPS and Galileo using satellite products from different analysis centers. GPS Solut. 2021;25(3). doi: 10.1007/s10291-021-01140-z

[34] Pan Y, Geng J, Liu K, Chen X, Fang R. Evaluation of rapid phase clock/bias products for PPP ambiguity resolution and its application to the M7.1 2019 Ridgecrest, California earthquake. Ad Space Res. 2020;65(11):2586–94. doi: 10.1016/j.asr.2020.02.016

[35] Geng J, Wen Q, Zhang Q, Li G, Zhang K. GNSS observable-specific phase biases for all-frequency PPP ambiguity resolution. J Geod. 2022;96(2). doi: 10.1007/s00190-022-01602-3

[36] Geng J, Zhang Q, Li G, Liu J, Liu D. Observable-specific phase biases of Wuhan multi-GNSS experiment analysis center’s rapid satellite products. Satell Navig. 2022;3(1). doi: 10.1186/s43020-022-00084-0

[37] Schaer S. Sinex bias—Solution (Software/technique) INdependent EXchange Format for GNSS Biases Version 1.00. 2015.[cited 12 Oct 2023]. Available: http://ftp.aiub.unibe.ch/bcwg/format/sinex_bias_100.pdf

[38] Schaer S, Villiger A, Arnold D, Dach R, Prange L, Jäggi A. The CODE ambiguity-fixed clock and phase bias analysis products: generation, properties, and performance. J Geod. 2021;95(7). doi: 10.1007/s00190-021-01521-9

[39] Schaer S. Bias-SINEX Format and Implications for IGS Bias Products. 2016.[cited 12 Oct 2023]. Available:http://acc.igs.org/workshop2016/presentations/Plenary_01_03.pdf

[40] Geng J, Yang S, Guo J. Assessing IGS GPS/Galileo/BDS-2/BDS-3 phase bias products with PRIDE PPP-AR. Satell Navig. 2021;2(1). doi: 10.1186/s43020-021-00049-9

[41] Vázquez-Ontiveros JR, Padilla-Velazco J, Gaxiola-Camacho JR, Vázquez-Becerra GE. Evaluation and Analysis of the Accuracy of Open-Source Software and Online Services for PPP Processing in Static Mode. Remote Sensing. 2023;15(8):2034. doi: 10.3390/rs15082034

[42] Geng J, Chen X, Pan Y, Zhao Q. A modified phase clock/bias model to improve PPP ambiguity resolution at Wuhan University. J Geod. 2019;93(10):2053–67. doi: 10.1007/s00190-019-01301-6

[43] Banville S, Geng J, Loyer S, Schaer S, Springer T, Strasser S. On the interoperability of IGS products for precise point positioning with ambiguity resolution. J Geod. 2020;94(1). doi: 10.1007/s00190-019-01335-w

[44] International gnss service (igs). IGS switch to IGS20/igs20.atx and repro3 standards – International GNSS Service. [cited 12 Oct 2023]. Available: https://igs.org/news/igs20/

[45] Guidelines For Long Product Filenames in the IGS. [cited 12 Oct 2023]. Available: https://files.igs.org/pub/resource/guidelines/Guidelines_For_Long_Product_Filenames_in_the_IGS_v2.0.pdf

[46] Romero I, Steigenberger P, Montenbruck O. Long Product Filenames in the IGS v1.0. 2019.[cited 12 Oct 2023]. Available: http://www.acc.igs.org/repro3/Long_Product_Filenames_v1.0.pdf

[47] Loyer S, Banville S, Perosanz F, Mercier F. Disseminating GNSS attitude for improved clock correction consistency. 2017. [cited 12 Oct 2023]. Available: https://igsac-cnes.cls.fr/documents/meeting/Poster_WS_IGS2017_fin.pdf

[48] Loyer S, Banville S, Geng J, Strasser S. Exchanging satellite attitude quaternions for improved GNSS data processing consistency. Advances in Space Research. 2021;68(6):2441–52. doi: 10.1016/j.asr.2021.04.049

[49] CDDIS | archive | gnss |[cited 12 Oct 2023]. https://cddis.nasa.gov/archive/gnss/products/

[50] IGS Data Center of Wuhan University. [cited 12 Oct 2023]. Available:http://www.igs.gnsswhu.cn/ftp://igs.gnsswhu.cn/pub/whu/phasebias

[51] Ogutcu S, Alcay S, Ozdemir BN, Li P, Zhang Y, Konukseven C, et al. Assessing the performance of BDS-3 for multi-GNSS static and kinematic PPP-AR. Advances in Space Research. 2023;71(3):1543–57. doi: 10.1016/j.asr.2022.10.016

[52] Zhou F, Cao X, Ge Y, Li W. Assessment of the positioning performance and tropospheric delay retrieval with precise point positioning using products from different analysis centers. GPS Solut. 2019;24(1). doi: 10.1007/s10291-019-0925-0

[53] Zhou F, Dong D, Li W, Jiang X, Wickert J, Schuh H. GAMP: An open-source software of multi-GNSS precise point positioning using undifferenced and uncombined observations. GPS Solut. 2018;22(2). doi: 10.1007/s10291-018-0699-9

[54] Héroux P, Kouba J. GPS precise point positioning using IGS orbit products. Physics and Chemistry of the Earth, Part A: Solid Earth and Geodesy. 2001;26(6–8):573–8. doi: 10.1016/s1464-1895(01)00103-x

[55] Ren Z, Lyu D, Gong H, Peng J, Huang X, Sun G. Continuous time and frequency transfer using robust GPS PPP integer ambiguity resolution method. GPS Solut. 2023;27(2). doi: 10.1007/s10291-023-01420-w

[56] Caswell TA, Droettboom M, Lee A, Andrade ES de, Hoffmann T, Klymak J, et al. matplotlib/matplotlib: REL: v3.5.2. Zenodo; 2022. doi: 10.5281/zenodo.6513224

[57] Elson P, Andrade ES Lucas G, May R, Hattersley R, Campbell E, et al. SciTools/cartopy: v0.20.0. Zenodo; 2021. doi: 10.5281/zenodo.5513855

[58] Geng J, Chen X, Pan Y, Mao S, Li C, Zhou J, et al. PRIDE PPP-AR: an open-source software for GPS PPP ambiguity resolution. GPS Solut. 2019;23(4). doi: 10.1007/s10291-019-0888-1

[59] Geng J, Mao S. Massive GNSS Network Analysis Without Baselines: Undifferenced Ambiguity Resolution. JGR Solid Earth. 2021;126(10). doi: 10.1029/2020jb021558

[60] PRIDELAB IN GNSS CENTER, Wuhan University. [cited 12 Oct 2023]. Available: http://pride.whu.edu.cn/indexone.shtml

[61] Ge M, Gendt G, Dick G, Zhang FP. Improving carrier-phase ambiguity resolution in global GPS network solutions. J Geodesy. 2005;79(1–3):103–10. doi: 10.1007/s00190-005-0447-0

